# The relationships between harsh physical punishment and child maltreatment in childhood and intimate partner violence in adulthood

**DOI:** 10.1186/s12889-017-4359-8

**Published:** 2017-05-23

**Authors:** Tracie O. Afifi, Natalie Mota, Jitender Sareen, Harriet L. MacMillan

**Affiliations:** 1 Departments of Community Health Sciences and Psychiatry, University of Manitoba, S113-750 Bannatyne Avenue, Winnipeg, MB R3E 0W5 Canada; 2 Department of Clinical Health Psychology, University of Manitoba, Winnipeg, Canada; 3 Departments of Psychiatry, Psychology, and Community Health Sciences, University of Manitoba, Winnipeg, Canada; 4 Department of Psychiatry and Behavioural Neurosciences, McMaster University, Hamilton, Canada; 5 Department of Pediatrics, McMaster University, Hamilton, Canada

**Keywords:** Child abuse, Child neglect, Physical abuse, Sexual abuse, Intimate partner violence, Physical punishment, And family violence

## Abstract

**Background:**

Physical punishment of children is an important public health concern. Yet, few studies have examined how physical punishment is related to other types of child maltreatment and violence across the lifespan. Therefore, the objective of the current study was to examine if harsh physical punishment (i.e., being pushed, grabbed, shoved, hit, and/or slapped without causing marks, bruises, or injury) is associated with an increased likelihood of more severe childhood maltreatment (i.e., physical abuse, emotional abuse, sexual abuse, physical neglect, emotional neglect, and exposure to intimate partner violence (IPV)) in childhood and perpetration or victimization of IPV in adulthood.

**Methods:**

Data were drawn from the National Epidemiologic Survey on Alcohol and Related Conditions collected in 2004 to 2005 (*n* = 34,402, response rate = 86.7%), a representative United States adult sample.

**Results:**

Harsh physical punishment was associated with increased odds of childhood maltreatment, including emotional abuse, sexual abuse, physical abuse, physical neglect, emotional neglect, and exposure to IPV after adjusting for sociodemographic factors, family history of dysfunction, and other child maltreatment types (range 1.6 to 26.6). Harsh physical punishment was also related to increased odds of experiencing IPV in adulthood (range 1.4 to 1.7).

**Conclusions:**

It is important for parents and professionals working with children to be aware that pushing, grabbing, shoving, hitting, or slapping children may increase the likelihood of emotional abuse, sexual abuse, physical abuse, physical neglect, emotional neglect, and exposure to IPV in childhood and also experiencing IPV victimization and/or perpetration in later adulthood.

## Background

Child maltreatment including physical abuse, emotional abuse, sexual abuse, physical neglect, emotional neglect, and exposure to intimate partner violence (IPV) as well as IPV in adulthood are forms of violence that have shown consistent associations with poor mental and physical health outcomes [1–18]. Child maltreatment and IPV jeopardizes the health of all family members, which significantly impacts communities and societies. It is known that an intergenerational cycle of child maltreatment exists for some families, meaning that those who were maltreated as children are more likely to maltreat to their own children [19–22]. It is also known that different types of child maltreatment commonly co-occur [5, 23–25]. However, it is currently unknown if the use of physical punishment (i.e., a physical act by a parent or guardian that causes deliberate pain in response to unwanted child behaviour) or harsh physical punishment (i.e., pushing, grabbing, shoving, hitting, and slapping) is related to more severe childhood maltreatment and to later violence in intimate relationships in adulthood. From a public health perspective, understanding this potential relationship is important because physical punishment remains common and is legal in North America [26–29]. This is the case even though 52 countries or states worldwide have banned all forms of physical punishment of children in all settings including home and schools [30]. Further, physical punishment has been found to be ineffective and related to numerous adverse health, behavioural, cognitive, and developmental outcomes [31–41]. Extending our knowledge in this area may be important for informing child maltreatment and violence prevention efforts and improving health across the lifespan both in countries with and without legal bans on the physical punishment of children.

Some researchers have examined the co-occurrence of physical punishment and more severe types of child maltreatment. For example, abusive parents have been found to be more likely to use physical punishment compared to non-abusive parents [42]. Similarly, Canadian child protection services data indicates that physical punitive violence was involved in 75% of substantiated cases of physical abuse as well as 13% of emotional maltreatment, 2% of sexual abuse, 2% of neglect, and 1% of exposure to IPV [43]. In addition, in a representative sample from North and South Carolina, physical punishment was associated with a 2.7 fold increase in the likelihood of physical abuse [44]. It may be that in some cases physical punishment as a disciplinary measure is a precursor in the progression and escalation towards child physical abuse; perhaps hitting as a disciplinary means evolves into more severe physical acts of maltreatment related to increasing anger in the person committing such acts (typically a parent) [45].

The current literature has important limitations. Many of the previous studies in the area have the used small and unrepresentative samples, which limits generalizability of the study findings. Additionally, studies have mainly focused on the relationship between physical punishment and only one type of child maltreatment – child physical abuse. However, it is possible that physical punishment may be associated with an increased likelihood of other child maltreatment types such as emotional abuse, emotional and physical neglect, sexual abuse, and exposure to IPV. We know from previous research that physical abuse commonly co-occurs with other types of child maltreatment [46, 47]. As well, physical punishment and child physical abuse both involve physical force and therefore, are believed to exist along a continuum rather than as distinct constructs [48–50]. Therefore, it is reasonable to hypothesize that physical punishment may also co-occur with other types of child maltreatment. Understanding this relationship has significant implications for child maltreatment prevention strategies. Importantly, it remains unknown if physical acts such as pushing, grabbing, shoving, hitting, or slapping are associated with other types of child maltreatment in a representative sample.

Furthermore, experiencing physical maltreatment in childhood with or without other forms of child maltreatment may make physical violence seem acceptable and may increase the likelihood of violence continuing into intimate partner relationships in adulthood [41]. Previous research has found that a child maltreatment history is associated with increased odds of violence in an intimate relationship in adulthood [2, 51]. It is possible that a similar association also exists for harsh physical punishment and violence in adult intimate relationships. Some support for this relationship has been found with research indicating a significant association between being physically punished as a teenager and increased odds of violence towards women in adulthood [52]. As well, research has indicated that maltreated children were more likely to be victims of IPV in adulthood [53]. Although these studies have examined child maltreatment and IPV in adulthood, our understanding of the association pushing, grabbing, shoving, hitting, and slapping children and later IPV in adulthood is limited. This identifies another important gap in knowledge; it is currently unknown if harsh physical punishment in childhood is linked with an increased likelihood of perpetration, victimization, and reciprocal violence in intimate adult relationships and if these associations exist independent of more severe child maltreatment.

The main objectives of the current research were to 1) examine if harsh physical punishment (i.e., pushing, grabbing, shoving, hitting, and/or slapping) is associated with increased odds of child maltreatment in childhood, including emotional abuse emotional neglect, sexual abuse, physical neglect, physical abuse, and exposure to IPV and 2) examine if harsh physical punishment is associated with increased odds of perpetration, victimization, or reciprocal IPV in adulthood in a large representative United States (US) sample after adjusting for possible confounding effects of sociodemographic variables, other types of child maltreatment, and a family history of dysfunction.

## Methods

### Survey

The current study examined data from Wave 2 of the National Epidemiologic Survey on Alcohol and Related Conditions (NESARC, study sample size *n* = 34,402). The NESARC is a nationally representative survey of the adult population in all 50 US states sponsored by the National Institutes of Health, the US Department of Health and Human Services, and the National Institute on Alcohol Abuse and Alcoholism. Respondents for the survey were randomly selected using a multistage stratified design in which primary sampling units were stratified according to specific sociodemographic criteria [54, 55]. Wave 2 was collected between 2004 and 2005 with the assistance of trained lay interviewers administering questions in the households of individuals aged 20 years and older (response rate: 86.7%). Data were collected using face-to-face, computer-assisted personal interviews conducted in respondents’ homes. The respondents were informed about the nature of the survey, the statistical use of the survey data, the voluntary aspects of their participation, and the Federal laws protecting confidentiality. Interviews were conducted after respondents received this information and provided consent to participate. The US Census Bureau and US Office of Management and Budget reviewed the research protocol and provided full ethical approval [56]. A thorough discussion of the NESARC and its survey design can be found elsewhere [57].

### Measures

#### Child maltreatment

The assessment of child maltreatment in the NESARC was based on items used in the Adverse Childhood Experiences (ACE) Study [58, 59]. These items were derived from both the Conflict Tactics Scale [60, 61], and the Childhood Trauma Questionnaire [62]. Emotional abuse, sexual abuse, physical neglect, physical abuse, and exposure to IPV before 18 years of age were measured using a 5-point ordinal scale to assess the frequency of the occurrence of each experience, ranging from “never” to “very often”. Emotional neglect before 18 years of age was measured using a 5-point scale that ranged from “never true” to “very often true”.

Exposure to harsh physical punishment was assessed using one item asking respondents how often they had been “pushed, grabbed, shoved, hit, or slapped” before they were 18 years of age by a parent or another adult in the household. Responses of “sometimes” or greater were coded as yes and responses of “never” or “rarely” were coded as no. This measure has been used in previous studies [3, 4, 63].

Physical abuse was measured using one item asking if the respondent had been hit so hard that it left marks, bruises, or resulted in injury. Responses of “sometimes” or greater were coded as yes and responses of “never” or “rarely” were coded as no. Physical neglect was assessed using four items and was defined as reporting any of the following experiences: being left alone before the age of 10 years, or going without needed resources such as clothing, shoes, materials for school, meals, or medical care when sick or injured. Sexual abuse was measured with four questions inquiring about touching or fondling and attempted or actual sexual intercourse by an adult or other individual that was unsolicited by the respondent or that happened before the respondent could understand what was occurring. Responses other than “never” indicated exposure to child sexual abuse. Emotional abuse was assessed with three items, including having had a parent or another adult in the household swear at, insult, or say things that were hurtful towards the respondent; threaten to hit or throw an object at them; or any other act that left the participant scared that they would be hurt. Respondents were identified as having experienced emotional abuse if they endorsed any of these items “fairly often” or “often”. Emotional neglect was assessed using five items defined as any response other than “never true” for the following: feelings of being in a close-knit family, feeling important or special by a family member, feeling like a family member believed in the respondent and provided them with strength or support, or having a member want them to succeed. Exposure to IPV as a child was assessed using four items to assess IPV against the respondents mother, which included being: (1) pushed, grabbed, slapped, or thrown something at their mother, (2) kicked, bit, or hit their mother with a fist or something hard, (3) repeatedly hit their mother for at least a few minutes, or (4) threatened her with a knife or gun, or use a knife or gun to hurt her.

#### Intimate partner violence (IPV)

A modified version of the Conflict Tactics Scale was used to assess IPV in the past year among survey participants who were in a relationship [64]. In the current sample, 80.7% were married, dating, or involved in a romantic relationship in the past year (19.0% indicated no and 0.3% indicated unknown). Those participants in a relationship differed from those not in a relationship with regard to sex, ethnicity, household income, education, and age. More specifically, females compared to males were less likely to be in a relationship. Black respondents were less likely and Asian/Native Hawaiian/Pacific Islander and Hispanic respondents were more likely to be in a relationship compared to white respondents. Respondents with higher levels of income and education were more likely to be in a relationship. Increasing age was associated with decreased likelihood of being in a relationship. Perpetration was assessed by asking respondents about the frequency of occurrence of the following six behaviors committed against their partner or spouse: 1) pushing, grabbing, or shoving, 2) slapping, kicking, biting, or hitting, 3) threats with a weapon (e.g., knife or gun), 4) cutting or bruising, 5) injuring their spouse or partner to the point that they needed medical care, and 6) forcing sexual intercourse. Victimization was assessed by asking respondents how often in the past year their spouse or partner had engaged in each of these six behaviors with them (i.e., never, once, 2 to 3 times, once a month, and more than once a month). For each behavior, a response of one time or more was coded as the experience being present. Responses to all items were used to create a four-level variable of mutually exclusive groups: no IPV, perpetration only, victimization only, and both perpetration and victimization.

#### Family history of dysfunction

Items pertaining to a family history of dysfunction before 18 years of age came from items adapted from the Adverse Childhood Experiences (ACE) Study [58, 59]. A dichotomous family history of dysfunction variable was created that categorized respondents endorsing one or more of the following events into the ‘yes’ category: a parent or other adult in the home (1) having problematic drinking or drug use; (2) going to jail/prison; (3) being treated or experienced hospitalization for psychiatric reasons; (4) having a suicide attempt; or (5) dying by suicide.

#### Sociodemographic covariates

The sociodemographic variables that were included in the logistic regression models included age (in years), sex (male or female), marital status, race/ethnicity, highest educational attainment, and past year household income in US dollars. Marital status was categorized into three groups: married/living together as common law, separated/divorced/widowed, and single/never married. Race/ethnicity was categorized into five groups: Hispanic, non-Hispanic White, non-Hispanic Black, non-Hispanic American Indian/Alaska Native, and non-Hispanic Asian/Hawaiian/other Pacific Islander. Highest educational attainment was measured in categories as less than high school, high school or equivalent, and some college or higher. Household income categories included up to $19,999; $20,000 to $39,999; $40,000 to $59,999; and more than $60,000. Table 1 presents the sociodemographic characteristics of the study sample. The majority of the sample was married or living in a common-law relationship (63.8%), White (70.9%), and over a third of the sample reported a household income in the past year of $60,000 or higher (37.8%).

**Table 1:** Sociodemographic characteristics of the study sample (*n* = 34,402)

	n (%) or Mean (SE)
Sex	
Males	14,458 (47.9%)
Females	19,944 (52.1%)
Race/Ethnicity	
White, non-Hispanic	20,034 (71.0%)
Black, non-Hispanic	6542 (11.0%)
American Indian/Alaska Native	570 (2.2%)
Asian/ Native Hawaiian/Other Pacific Islander	948 (4.2%)
Hispanic	6308 (11.6%)
Household income (Past Year)	
$0–$19,999	7938 (18.5%)
$20,000–$39,999	8828 (24.3%)
$40,000–$59,999	6403 (19.4%)
$60,000+	11,233 (37.9%)
Marital status	
Married/co-habiting	18,751 (63.8%)
Widowed/divorced/separated	9063 (18.8%)
Never married	6588 (17.4%)
Education	
Less than high school	5450 (14.0%)
High school or equivalent	9376 (27.5%)
Some college or higher	19,576 (56.6%)
Age	48.1 (0.17)

All n’s were unweighted. All percents were weighted

### Statistical analyses

Wave 2 statistical weights were provided in the dataset and were computed to reflect design characteristics of the NESARC and to account for non-response and sample attrition [65]. Statistical weights were applied in all analyses. Taylor series linearization was performed as a variance estimation technique to account for the complex survey design. First, logistic regression models were computed to examine the associations between harsh physical punishment and more severe child maltreatment. Models were adjusted for sociodemographic variables, co-occurrence of other types of childhood maltreatment, and family history of dysfunction. Multinomial logistic regression analyses were used to examine relationships between harsh physical punishment and IPV, adjusting for the same covariates as in the previous analyses.

## Results

Among the respondents, 16.7% reported that they had experienced harsh physical punishment including being pushed, grabbed, shoved, hit, and/or slapped before the age of 18 by a parent or another adult in the household. Tables 2 and 3 presents the prevalence and the relationships between harsh physical punishment and child maltreatment. Harsh physical punishment was associated with an increased likelihood of having experienced all types of child maltreatment (adjusted odds ratio [AOR] range 1.6–26.6). Table 4 presents the relationships between harsh physical punishment and IPV in adulthood. The findings indicate that harsh physical punishment was associated with an increased likelihood of IPV perpetration, victimization, and reciprocal IPV (AOR range 1.4–1.7).

**Table 2:** Prevalence of child maltreatment and adult IPV among individuals with and without harsh physical punishment in childhood

	No harsh physical punishment (pushing, grabbing, shoving, hitting, and/or slapping) n (%)	Harsh physical punishment (pushing, grabbing, shoving, hitting, and/or slapping) n (%)
Child maltreatment		
Emotional abuse		
n (%)	638 (2.1)	2268 (36.8)
Sexual abuse		
n (%)	2248 (7.4)	1602 (25.0)
Physical neglect		
n (%)	5411 (18.6)	3133 (51.6)
Emotional neglect		
n (%)	1892 (6.2)	1509 (24.6)
Physical abuse		
n (%)	256 (0.8)	2451 (39.0)
Exposure to IPV		
n (%)	1748 (5.5)	2061 (32.9)
Any child maltreatment		
n (%)	8838 (30.1)	4754 (78.2)
Intimate partner violence (IPV)		
No IPV		
n (%)	19,359 (93.6)	3982 (86.1)
Perpetration only		
n (%)	456 (1.8)	191 (3.5)
Victimization only		
n (%)	397 (1.8)	185 (3.8)
Both perpetration and victimization		
n (%)	664 (2.8)	368 (6.7)

All n’s were unweighted. All percents were weighted

**Table 3:** Associations between harsh physical punishment in childhood and child maltreatment

	AOR (95% CI)						
	Emotional abuse model	Sexual abuse model	Physical neglect model	Emotional neglect model	Physical abuse model	Exposure to IPV model	Any child maltreatment model
Harsh physical punishment	7.6 (6.6, 8.9)***	1.7 (1.5, 1.9)***	1.8 (1.6, 2.0)***	1.6 (1.3, 1.8)***	26.6 (21.6, 32.6)***	2.6 (2.2, 3.0)***	7.0 (6.4, 7.5)***
Sex	1.0 (0.9, 1.2)	3.3 (3.0, 3.7)***	0.7 (0.6, 0.7)***	1.2 (1.0, 1.3)*	0.9 (0.8, 1.0)	1.3 (1.1, 1.5)***	1.0 (1.0, 1.1)
Age	1.00 (1.0, 1.0)***	1.0 (1.0, 1.0)***	1.0 (1.0, 1.0)	1.0 (1.0, 1.0)***	1.0 (1.0, 1.0)	1.0 (1.0, 1.0)*	1.0 (1.0, 1.0)**
Marital status							
Widowed/divorced/separated	1.3 (1.1, 1.6)**	1.2 (1.1, 1.4)**	1.0 (0.9, 1.1)	1.1 (1.0, 1.3)	1.1 (0.9, 1.2)	0.9 (0.8, 1.0)	1.1 (1.0, 1.2)**
Never Married	1.2 (1.0, 1.5)	1.0 (0.9, 1.2)	1.1 (1.0, 1.2)	0.9 (0.7, 1.0)*	0.6 (0.5, 0.8)***	0.7 (0.6, 0.8)***	0.9 (0.8, 1.0)*
Race/Ethnicity							
Black, Non, Hispanic	0.8 (0.7, 1.0)	1.1 (1.0, 1.3)	1.0 (0.9, 1.1)	0.7 (0.5, 0.8)***	1.7 (1.4, 2.1)***	1.7 (1.5, 1.9)***	1.1 (1.0, 1.3)*
American Indian/Alaska Native	0.8 (0.5, 1.3)	1.4 (1.0, 1.9)*	1.1 (0.9, 1.4)	1.0 (0.7, 1.3)	1.1 (0.8, 1.7)	1.4 (0.9, 2.0)	1.2 (1.0, 1.5)
Hawaiian/Other Pacific Islander	0.7 (0.5, 1.2)	0.5 (0.4, 0.7)***	1.7 (1.4, 2.1)***	1.2 (0.8, 1.7)	1.1 (0.7, 1.8)	1.7 (1.2, 2.3)**	1.4 (1.2, 1.7)***
Hispanic	0.6 (0.5, 0.7)***	0.9 (0.8, 1.1)	1.4 (1.2, 1.6)***	1.3 (1.1, 1.5)**	1.1 (0.9, 1.4)	1.4 (1.2, 1.6)***	1.3 (1.1, 1.5)***
Education							
High school or equivalent	1.1 (0.8, 1.3)	1.2 (1.0, 1.4)	0.9 (0.8, 1.00)*	0.8 (0.7, 0.9)**	0.9 (0.8, 1.2)	0.9 (0.7, 1.1)	0.8 (0.7, 0.9)***
Some college or higher	1.2 (0.9, 1.5)	1.4 (1.1, 1.6)***	1.0 (0.9, 1.2)	0.6 (0.5, 0.7)***	0.8 (0.7, 1.1)	0.7 (0.6, 0.8)***	0.8 (0.8, 0.9)**
Household income							
$20,000, $39,999	0.8 (0.7, 1.0)	1.0 (0.9, 1.2)	0.9 (0.8, 1.0)*	0.8 (0.7, 0.9)**	0.9 (0.7, 1.1)	1.0 (0.9, 1.2)	0.9 (0.8, 0.9)**
$40,000, $59,999	0.9 (0.7, 1.1)	0.9 (0.8, 1.1)	0.9 (0.8, 1.0)*	0.8 (0.7, 1.0)*	0.9 (0.7, 1.1)	0.9 (0.8, 1.1)	0.8 (0.7, 0.9)***
$60,000+	0.9 (0.7, 1.1)	0.9 (0.8, 1.1)	0.9 (0.8, 1.0)*	0.6 (0.5, 0.7)***	0.8 (0.6, 0.9)*	0.8 (0.7, 1.0)*	0.7 (0.7, 0.8)***
Any family history of dysfunction	1.6 (1.4, 1.9)***	1.8 (1.6, 2.0)***	1.9 (1.7, 2.0)***	1.5 (1.4, 1.7)***	1.2 (1.0, 1.4)*	5.3 (4.7, 6.1)***	3.3 (3.0, 3.5)***
Sexual abuse	1.9 (1.6, 2.2)***	N/A	2.0 (1.8, 2.3)***	1.4 (1.3, 1.6)***	1.6 (1.4, 2.0)***	1.7 (1.5, 2.0)***	N/A
Physical neglect	2.1 (1.8, 2.4)***	2.0 (1.8, 2.3)***	N/A	2.2 (1.9, 2.4)***	1.9 (1.6, 2.2)***	2.3 (2.02.6)***	N/A
Emotional neglect	3.0 (2.6, 3.5)***	1.4 (1.2, 1.6)***	2.1 (1.9, 2.4)***	N/A	1.7 (1.5, 2.0)***	1.3 (1.1, 1.5)***	N/A
Emotional abuse	N/A	1.7 (1.4, 2.0)***	2.0 (1.7, 2.3)***	2.9 (2.5, 3.4)***	4.3 (3.6, 5.1)***	1.9 (1.5, 2.3)***	N/A
Physical abuse	4.0 (3.4, 4.8)***	1.4 (1.3, 1.6)**	1.8 (1.5, 2.0)***	1.5 (1.3, 1.8)***	N/A	1.8 (1.5, 2.1)***	N/A
Exposure to IPV	1.9 (1.6, 2.3)***	1.7 (1.5, 1.9)***	2.2 (2.0, 2.5)***	1.3 (1.1, 1.5)**	2.1 (1.8, 2.5)***	N/A	N/A

Adjusted odds ratio (*AOR*). Adjusted for sex, age, marital status, race/ethnicity, education, and household income, any family history of dysfunction, and each other type of childhood maltreatment. Sex reference group was males. Marital status reference group is married/co-habiting. Race/ethnicity reference group is White, non-Hispanic. Education reference group is less than high school. Household income reference group is $0–$19,999. *p ≤ .05, **p ≤ .01, ***p ≤ .001

**Table 4:** Associations between harsh physical punishment in childhood and intimate partner violence in adulthood

	Perpetration only	Victimization only	Both perpetration and victimization
Harsh physical punishment	1.7 (1.4, 2.0)***	1.7 (1.4, 2.1)***	1.4 (1.1, 1.7)*
Sex	1.2 (1.0, 1.4)*	2.7 (2.1, 3.3)***	0.5 (0.4, 0.6)***
Age	1.0 (1.0, 1.0)***	1.0 (1.0, 1.0)***	1.0 (1.0, 1.0)***
Marital status			
Widowed/divorced/separated	1.1 (0.9, 1.4)	0.7 (0.5, 0.9)*	2.7 (2.1, 3.6)***
Never Married	0.8 (0.7, 1.0)	0.7 (0.5, 1.0)*	1.3 (1.0, 1.7)
Race/Ethnicity			
Black, Non, Hispanic	2.0 (1.7, 2.4)***	2.0 (1.6, 2.5)***	1.7 (1.3, 2.2)***
American Indian/Alaska Native	1.6 (1.0, 2.5)*	1.9 (1.1, 3.2)*	1.0 (0.5, 2.1)
Hawaiian/Other Pacific Islander	1.1 (0.7, 2.0)	0.9 (0.5, 1.8)	1.1 (0.4, 2.9)
Hispanic	1.2 (0.9, 1.4)	1.2 (0.9, 1.6)	1.4 (1.0, 1.8)*
Education			
High school or equivalent	0.9 (0.7, 1.1)	1.3 (0.9, 1.8)	1.2 (0.9, 1.7)
Some college or higher	0.7 (0.5, 0.9)**	0.9 (0.6, 1.2)	1.3 (0.9, 1.8)
Household income			
$20,000–$39,999	0.9 (0.7, 1.1)	0.7 (0.6, 1.0)*	0.8 (0.6, 1.1)*
$40,000–$59,999	0.7 (0.6, 0.9)**	0.6 (0.4, 0.8)**	0.6 (0.4, 0.9)*
$60,000+	0.6 (0.5, 0.8)***	0.5 (0.4, 0.7)***	0.7 (0.5, 1.0)
Any family history of dysfunction	1.3 (1.1, 1.6)**	1.2 (0.9, 1.5)	1.8 (1.5, 2.2)***
Any child maltreatment	2.1 (1.7, 2.6)***	1.5 (1.2, 1.9)***	1.9 (1.5, 2.5)***

AOR. Adjusted for sex, age, marital status, race/ethnicity, education, and household income, any family history of dysfunction, and any childhood maltreatment. Sex reference group was males. Marital status reference group is married/co-habiting. Race/ethnicity reference group is White, non-Hispanic. Education reference group is less than high school. Household income reference group is $0–$19,999. Perpetration only, victimization only, and both perpetration and victimization are mutually exclusive groups. *p ≤ .05, **p ≤ .01, ***p ≤ .001

## Discussion

This study has two main important findings. First, harsh physical punishment was associated with increased odds of all child maltreatment types including emotional abuse, sexual abuse, physical abuse, physical neglect, emotional neglect, and exposure to IPV after adjusting for sociodemographic factors, family history of dysfunction (AOR range 1.6 to 26.6). Second, harsh physical punishment was associated with increased odds of IPV perpetration, victimization, and reciprocal violence in adulthood (AOR range 1.4 to 1.7) in adjusted models.

Previous research has indicated that physical punishment is associated with an increased likelihood of physical abuse [44, 66]. Our findings show this association as well, but also indicate that the relationship with harsh physical punishment extends beyond physical abuse to include an increased likelihood of childhood emotional abuse, sexual abuse, physical neglect, emotional neglect, and exposure to IPV, as well as perpetration, victimization, and reciprocal IPV in adult relationships. Importantly, the relationships between harsh physical punishment and each individual child maltreatment type were not accounted for by experiencing other types of child maltreatment. The strongest effects were noted between harsh physical punishment and physical abuse, emotional abuse, and exposure to IPV. The effect for physical neglect, emotional neglect, sexual abuse, IPV perpetration, IPV victimization, and reciprocal IPV had lower effect sizes, but all remained statistically significant.

This study has several limitations including the cross-sectional nature of the data, which precludes making causal inferences in the relationship between harsh physical punishment and child maltreatment and IPV in adulthood. This is especially the case when examining the relationships between harsh physical punishment and child maltreatment because the temporal nature of these relationships cannot be determined with these data. Second, all data were based on self-reports from the respondent. Third, data on childhood experiences were assessed retrospectively, which may introduce some sampling error due to recall and reporting bias. However, evidence indicates that adverse childhood events can be accurately recalled [67]. Additionally, the current data collection used a common flashcard method where respondents viewed the flashcards and indicated with codes the traumatic events that had occurred. This means neither the interviewer nor the respondent had to verbally identify the event during the interview, which may increase the accuracy of reporting. Fourth, harsh physical punishment was assessed using one item. Physical acts of pushing, grabbing, shoving, hitting, and/or slapping are harsh and may go beyond what some would consider “mild disciplinary spanking.” Finally, the measure of parental psychopathology was limited because it was assessed using the respondent’s retrospective recall and knowledge of parental problems with alcohol or drugs and being treated or hospitalized for mental illness.

These findings have important implications for clinical practice, prevention, and policy. It is essential that professionals working with children and families are aware of the statistically significant co-occurrence of harsh physical punishment and physical abuse, sexual abuse, emotional abuse, emotional neglect, physical neglect, and exposure to IPV in childhood and also experiencing IPV victimization and/or perpetration in later adulthood. It might be that pushing, grabbing, shoving, hitting, and/or slapping children are risk indicators of poor parenting or discipline strategies linked with increased odds of child maltreatment for some families, and violence in intimate relationships in adulthood. Although a causal role for harsh physical punishment and child maltreatment in relation to violence in intimate adult relationships cannot be determined with these data, health care providers should consider the co-occurrence of these experiences and the possibility of escalating and continuation of violence when making recommendations regarding physical discipline. Based on findings from earlier studies together with these new results, it is recommended that parents or other adult caregivers should not physically punish children. This recommendation is made in an effort to protect children from potentially harmful forms of physical punishment and to reduce the likelihood of exposure to more severe forms of child maltreatment and violence in intimate adult relationships. We also know from previous research that child maltreatment and IPV is associated with poor mental and physical health outcomes [1–11, 13–18, 68]. This adds to the importance and urgency of understanding how we can successfully reduce and prevent exposure to child maltreatment and violence across the lifespan.

## Conclusions

From a public health perspective, it is a priority to prevent children’s exposure to maltreatment during childhood as well as IPV in adulthood. Preventing or reducing the prevalence of child maltreatment and IPV is a challenging task. With regard to prevention of IPV in adulthood, the focus has mainly been on education about healthy relationships and prevention of dating violence [69]. However, for some individuals, exposure to harsh physical punishment may set the stage for acceptance regarding the use of physical force in relationships and establish some degree of tolerance of violence perpetration or victimization in an intimate relationship. Most of the research in this area has focused on the link between more severe child maltreatment and IPV [70]. Insufficient attention has been paid to the overlap between harsh physical punishment in childhood and IPV in adulthood and how this relationship can inform prevention strategies. Traditionally, research and policies have focused on reducing male violence against female partners. Another important strategy to examine is the role of both male and female caregivers in preventing IPV through reducing their use of harsh physical punishment of children. Positive parenting approaches that do not include physical discipline are important for reducing the likelihood that a child will experience physical punishment, child maltreatment, and IPV in adult intimate relationships.

## Abbreviations

ACE: Adverse Childhood Experiences
AOR: Adjusted odds ratio
AUDADIS-IV: Alcohol Use Disorder and Associated Disabilities Interview Schedule
DSM: Diagnostic and Statistical Manual of Mental Disorders
IPV: Intimate partner violence
NESARC: National Epidemiologic Survey on Alcohol and Related Conditions
US: United States

## References

[1] Enns MW, Cox BJ, Asmundson GJ, Stein MB, Sareen J, Afifi TO (2008). Population attributable fractions of psychiatric disorders and suicide ideation and attempts associated with adverse childhood experiences. Am J Public Health.

[2] MacMillan H, Cox BJ, Asmundson GJG, Stein MB, Sareen J, Afifi TO (2009). Mental health correlates of intimate partner violence in marital relationships in a nationally representative sample of males and females. J Interpers Violence.

[3] Mota N, MacMillan HL, Sareen J, Afifi TO (2013). Harsh physical punishment in childhood and adult physical health. Pediatrics.

[4] Afifi TTO, Mota NP, Dasiewicz P, MacMillan HHL, Sareen J, Straus M (2012). Physical punishment and mental disorders: results from a nationally representative US sample. Pediatrics.

[5] Mather A, Boman J, Fleisher W, Enns MW, Macmillan H, Afifi TO (2011). Childhood adversity and personality disorders: results from a nationally representative population-based study. J Psychiatr Res.

[6] MacMillan H, Boyle M, Taillieu T, Cheung K, Sareen J, Afifi TO (2014). Child abuse and mental disorders in Canada. CMAJ.

[7] Brownridge DA, Cox BJ, Sareen J, Afifi TO (2006). Physical punishment, childhood abuse and psychiatric disorders. Child Abuse Negl.

[8] Beydoun HA, Beydoun MA, Kaufman JS, Lo B, Zonderman AB (2012). Intimate partner violence against adult women and its association with major depressive disorder, depressive symptoms and postpartum depression: a systematic review and meta-analysis. Soc Sci Med.

[9] Bruffaerts R, Demyttenaere K, Borges G, Haro JM, Chiu WT, Hwang I (2010). Childhood adversities as risk factors for onset and persistence of suicidal behaviour. Br J Psychiatry.

[10] Chen LP, Murad MH, Paras ML, Colbenson KM, Sattler AL, Goranson EN (2010). Sexual abuse and lifetime diagnosis of psychiatric disorders: systematic review and meta-analysis. Mayo Clin Proc.

[11] Kendler KS, Kuhn JW, Prescott CA (2004). Childhood sexual abuse, stressful life events and risk for major depression in women. Psychol Med.

[12] Kessler RC, Davis CG, Kendler KS (1997). Childhood adversity and adult psychiatric disorder in the US National Comorbidity Survey. Psychol Med.

[13] MacMillan HL, Boyle MH, Wong MY-Y, Duku EK, Fleming JE, Walsh CA (1999). Slapping and spanking in childhood and its association with lifetime prevalence of psychiatric disorders in a general population sample. CMAJ.

[14] Scott KM, Smith DR, Ellis PM. Prospectively ascertained child maltreatment and its association with DSM-IV mental disorders in young adults Arch Gen Psychiatry 2010;67:712–9. doi:10.1001/archgenpsychiatry.2010.71. 10.1001/archgenpsychiatry.2010.71 20603452

[15] Afifi TO, MacMillan H, Boyle MH, Cheung K, Taillieu T, Turner S, et al. Child abuse and physical health in Canada. Heal Reports. 2016. Under Review. 26983007

[16] Martin MS, Dykxhoorn J, Colman I, Afifi TO (2016). Child abuse and the prevalence of suicide attempts among those reporting suicide ideation. Soc Psychiatry Psychiatr Epidemiol.

[17] Taillieu TL, Brownridge DA, Sareen J, Afifi TO (2016). Childhood emotional maltreatment and mental disorders: results from a nationally representative adult sample from the United States. Child Abuse Negl.

[18] Turner S, Taillieu T, Cheung K, Fortier J, Afifi TO. Childhood sexual abuse and mental health outcomes among males: results from a nationally representative United States sample. Child Abuse Negl. 2017; In Press 10.1016/j.chiabu.2017.01.018 28185670

[19] Widom CS, Czaja SJ, DuMont KA (2015). Intergenerational transmission of child abuse and neglect: real or detection bias?. Science.

[20] Putnam-Hornstein E, Cederbaum JA, King B, Eastman AL, Trickett PK (2015). A population-level and longitudinal study of adolescent mothers and intergenerational maltreatment. Am J Epidemiol.

[21] Ertem IO, Leventhal JM, Dobbs S (2000). Intergenerational continuity of child physical abuse: how good is the evidence?. Lancet.

[22] Pears KC, Capaldi DM (2001). Intergenerational transmission of abuse: a two-generational prospective study of an at-risk sample. Child Abuse Negl.

[23] Dong M, Anda RF, Felitti VJ, Dube SR, Williamson DF, Thompson TJ (2004). The interrelatedness of multiple forms of childhood abuse, neglect, and household dysfunction. Child Abuse Negl.

[24] Clemmons JC, Walsh K, DiLillo D, Messman-Moore TL (2007). Unique and combined contributions of multiple child abuse types and abuse severity to adult trauma symptomatology. Child Maltreat.

[25] Edwards VJ, Holden GW, Felitti VJ, Anda RF (2003). Relationship between multiple forms of childhood maltreatment and adult mental health in community respondents: results from the adverse childhood experiences study. Am J Psychiatry.

[26] Straus MA, Stewart JH (1999). Corporal punishment by American parents: national data on prevalence, chronicity, severity, and duration, in relation to child and family characteristics. Clin Child Fam Psychol Rev.

[27] Clément M-È, Chamberland C (2014). Trends in corporal punishment and attitudes in favour of this practice: toward a change in societal norms. Can J Community Ment Heal.

[28] Lee SJ, Grogan-Kaylor A, Berger LM (2014). Parental spanking of 1-year-old children and subsequent child protective services involvement. Child Abuse Negl.

[29] Zolotor AJ, Robinson TW, Runyan DK, Barr RG, Murphy RA (2011). The emergence of spanking among a representative sample of children under 2 years of age in north Carolina. Front Psychiatry.

[30] Global Initiative to End Corporal Punishment of Children. States which have prohibited all corporal punishment of children. 2015. Retrieved from: http://www.endcorporalpunishment.org/.

[31] MacKenzie MJ, Nicklas E, Brooks-Gunn J, Waldfogel J (2014). Spanking and children’s externalizing behavior across the first decade of life: evidence for transactional processes. J Youth Adolesc.

[32] MacKenzie MJ, Nicklas E, Waldfogel J, Brooks-Gunn J (2013). Spanking and child development across the first decade of life. Pediatrics.

[33] MacKenzie MJ, Nicklas E, Waldfogel J, Brooks-Gunn J (2012). Corporal punishment and child behavioural and cognitive outcomes through 5 years of age: evidence from a contemporary urban birth cohort study. Infant Child Dev.

[34] Altschul I, Lee SJ, Gershoff ET (2016). Hugs, not hits: warmth and spanking as predictors of child social competence. J Marriage Fam.

[35] Lee SJ, Taylor CA, Altschul I, Rice JC (2013). Parental spanking and subsequent risk for child aggression in father-involved families of young children. Child Youth Serv Rev.

[36] Gershoff ET, Grogan-Kaylor A (2016). Spanking and child outcomes: old controversies and new meta-analyses. J Fam Psychol.

[37] Gershoff ET (2013). Spanking and child development: we know enough now to stop hitting our children. Child Dev Perspecitves.

[38] American Academy of Pediatrics (1998). Guidance for effective discipline. Pediatrics.

[39] American Academy of Child and Adolescent Psychiatry. Policy statement on corporal punishment. 2012. Retrieved from: https://www.aacap.org/aacap/Policy_Statements/2012/Policy_Statement_on_Corporal_Punishment.aspx

[40] Afifi TO, Ford D, Gershoff E, Merrick M, Grogan-Kaylor A, Ports KA, et al. Spanking and adult mental health impairment: The case for the designation of spanking as an adverse childhood experience. Child Abuse Negl. n.d.;In Press. 10.1016/j.chiabu.2017.01.014 PMC7983058 28126359

[41] Gershoff ET (2002). Corporal punishment by parents and associated child behaviors and experiences: a meta-analytic and theoretical review. Psychol Bull.

[42] Whipple EE, Richey CA (1997). Crossing the line from physical discipline to child abuse: how much is too much?. Child Abuse Negl.

[43] Durrant JE, Trocmé N, Barbara MCF, Black T, Knoke D. Punitive violence against children in Canada 2006:CECW information sheet #41E. Toronto. ON.

[44] Zolotor AJ, Theodore AD, Chang JJ, Berkoff MC, Runyan DK (2008). Speak softly and forget the stick: corporal punishment and child physical abuse. Am J Prev Med.

[45] Knox M (2010). On hitting children: a review of corporal punishment in the United States. J Pediatr Heal Care.

[46] Afifi TO, MacMillan HL, Taillieu T, Cheung K, Turner S, Tonmyr L, et al. Relationship between child abuse exposure and reported contact with child protection organizations: results from the Canadian community health survey. Child Abuse Negl. 2015; 10.1016/j.chiabu.2015.05.001 26002601

[47] Higgins DJ, McCabe MP (2001). Multiple forms of child abuse and neglect: adult retrospective reports. Aggress Violent Behav.

[48] Coontz PD, Martin JA. Understanding violent mothers and fathers: Assessing explanations offered by mothers and fathers of their use of control punishment. In: Hotaling GT, Finkelhor D, Kirkpatrick JT, Straus MA, editors. Fam. Abus. its consequences New Dir. Res., Thousand Oaks (CA): Sage Publications; 1988, p. 77–90.

[49] Dussich JP, Maekoya C (2007). Physical child harm and bullying-related behaviors: a comparative study in Japan, South Africa, and the United States. Int J Offender Ther Comp Criminol.

[50] Österman K, Björkqvist K, Wahlbeck K (2014). Twenty-eight years after the complete ban on the physical punishment of children in Finland: trends and psychosocial concomitants. Aggress Behav.

[51] McMahon K, Hoertel N, Wall MM, Okuda M, Limosin F, Blanco C (2015). Childhood maltreatment and risk of intimate partner violence: a national study. J Psychiatr Res.

[52] Straus MA, Kantor GK (1994). Corporal punishment of adolescents by parents: a risk factor in the epidemiology of depression, suicide, alcohol abuse, child abuse, and wife beating. Adolescence.

[53] Renner LM, Slack KS (2006). Intimate partner violence and child maltreatment: understanding intra- and intergenerational connections. Child Abuse Negl.

[54] Grant BF, Kaplan KD, Shepard J, Moore T. Source and accuracy statement for Wave 1 of the 2001–2002 National Epidemiologic Survey on Alcohol and Related Conditions. Bethesda, MD: National Institute on Alcohol Abuse and Alcoholism: 2003.

[55] Grant BF, Kaplan KD. Source and accuracy statement for Wave 2 of the 2004–2005 National Epidemiologic Survey on Alcohol and Related Conditions. Bethesda, MD: National Institute on Alcohol Abuse and Alcoholism: 2005.

[56] National Epidemiological Survey on Alcohol and Related Conditions (NESARC). Wave 2 NESARC data notes. 2008.

[57] Grant BF, Hasin DS, Stinson FS, Dawson DA, Ruan WJ, Goldstein RB (2005). Prevalence, correlates, co-morbidity, and comparative disability of DSM-IV generalized anxiety disorder in the USA: results from the National Epidemiologic Survey on alcohol and related Conditions. Psychol Med.

[58] Dong M, Anda RF, Dube SR, Giles WH, Felitti VJ (2003). The relationship of exposure to childhood sexual abuse to other forms of abuse, neglect, and household dysfunction during childhood. Child Abuse Negl.

[59] Dube SR, Felitti VJ, Dong M, Chapman DP, Giles WH, Anda RF (2003). Childhood abuse, neglect, and household dysfunction and the risk of illicit drug use: the adverse childhood experiences study. Pediatrics.

[60] Straus MA (1979). Measuring Intrafamily conflict and violence: the conflict tactics (CT) scales. J Marriage Fam..

[61] Straus MA, Hamby SL, Boney-McCoy S, Sugarman DB (1996). The revised conflict tactics scales (CTS2): development and preliminary psychometric data. J Fam Issues.

[62] Bernstein DP, Fink L, Handelsman L, Foote J, Lovejoy M, Wenzel K (1994). Initial reliability and validity of a new retrospetive measure of child abuse and neglect. Am J Psychiatry.

[63] Taillieu TL, Mota N, Keyes KM, Sareen J, Afifi TO (2014). Age, sex, and racial differences in harsh physical punishment: results from a nationally representative United States sample. Child Abuse Negl.

[64] Straus MA. Measuring intrafamily conflict and violence: The Conflict Tactics (CT) Scales., New Brunswick, NJ: Tranaction; 1990, p. 29–47.

[65] National Institute of Health. Alcohol use and alcohol use disorders in the United States: A 3-year follow-up: Main findings from the 2004–2005 Wave 2 National Epidemiologic Survey of Alcohol and Related Conditions (NESARC). 2010.

[66] Fréchette S, Zoratti M, Romano E (2015). What is the link between corporal punishment and child physical abuse?. J Fam Violence.

[67] Hardt J, Rutter M (2004). Validity of adult retrospective reports of adverse childhood experiences: review of the evidence. J Child Psychol Psychiatry.

[68] Kessler RC, Frank RG, Edlund M, Katz S, Lin E, Leaf P (1997). Differences in the use of psychiatric outpatient services between the United States and Ontario. N Engl J Med.

[69] Fellmeth GL, Heffernan C, Nurse J, Habibula S, Sethi D (2013). Education and skill-based interventions for preventing relationship and dating violence in adolescents and young adults. Cochrane Database Syst Rev.

[70] Millett LS, Kohl PL, Jonson-Reid M, Drake B, Petra M (2013). Child maltreatment victimization and subsequent perpetration of young adult intimate partner violence: an exploration of mediating factors. Child Maltreat..

